# 晚期肺鳞癌的治疗

**DOI:** 10.3779/j.issn.1009-3419.2016.10.10

**Published:** 2016-10-20

**Authors:** 以香 朱, 镨元 邢, 峻岭 李

**Affiliations:** 100021 北京，国家癌症中心/中国医学科学院北京协和医学院肿瘤医院 National Cancer Center/Cancer Hospital, Chinese Academy of Medical Sciences and Peking Union Medical College, Beijing 100021, China

**Keywords:** 肺肿瘤, 化疗, 靶向治疗, Lung neoplasms, Chemotherapy, Targeted therapy

## Abstract

肺癌是全世界肿瘤死亡的首要原因，肺鳞癌（squamous cell lung cancer, SQCLC）作为肺癌的一种常见的病理类型，全世界每年约40余万人因其致死。其常规治疗方法主要包括手术治疗、化学治疗和分子靶向治疗。但是大多数患者确诊的时候已经是晚期，失去了手术的机会。尽管分子靶向治疗在肺腺癌的治疗中具有里程碑式的作用，但是对肺鳞癌而言，尚无特异性的分子靶标药物，因此，对于晚期肺鳞癌的标准治疗仍是含铂双药方案。而大多数患者经历了一线、二线治疗失败后都面临无药可用的状态。本文旨在对晚期肺鳞癌的常规治疗进行系统性的综述，探讨晚期肺鳞癌的治疗方案以及发展方向。

## Abstract (en)

Lung cancer is the deadliest cancer in the worldwide. Non-small cell lung cancer (NSCLC) accounts for 85% of lung tumor diagnoses. Squamous cell lung cancer (SQCLC) is a common pathological type, almost 20%-30% of NSCLC. Surgery, chemotherapy, and molecular targeted therapies are the mainstay of treatment for patients with SQCLC. But most patients are diagnosed at advanced stage so that they miss the chance of operation. While noteworthy outcomes have improved with adenocarcinoma of lung with epidermal growth factor receptor tyrosine kinase inhibitors (EGFR-TKIs), a therapeutic plateau for advanced squamous cell lung cancer patients are still not solved. EGFR-TKIs are unsuitable for or mostly ineective in advanced SQCLC. Patients with advanced SQCLC ramain treated with platinum based chemotherapy. Tis reciew systematicly describe the treatment of squamous cell carcinoma of the lung.

肺癌仍是全球癌症发病率及癌症死亡率最高的恶性肿瘤^[1]^。在诊断的肺癌患者中，非小细胞肺癌（non-small cell lung cancer, NSCLC）约为85%，其中肺鳞癌约占20%-30%，是NSCLC中最常见的病理类型之一^[2]^。流行病学资料表明，其发病与吸烟密切相关^[3]^。而且，因早期症状不明显，大部分患者在确诊时已为晚期，失去手术机会。过去的十几年中，晚期肺鳞癌标准的一线治疗方案仍旧是含铂双药的化学治疗。尽管分子靶向治疗在晚期肺腺癌的治疗中取得了里程碑式的作用，如人表皮生长因子受体（epidermal growth factor receptor, EGFR）抑制剂吉非替尼、厄洛替尼和埃克替尼以及间变性淋巴瘤激酶（anaplastic lymphoma kinase, ALK）抑制剂克唑替尼、色瑞替尼等，临床研究证实该类药物在*EGFR*突变的患者中，客观缓解率约为50%，中位生存时间可延长10个月-11个月^[4, 5]^。但遗憾的是，分子靶向药物在晚期肺鳞癌患者中疗效并不明确，因此，晚期肺鳞癌的治疗效果远远不如肺腺癌。本文旨在综述近年来晚期肺鳞癌的治疗以及一些相关的临床研究，从肺鳞癌传统化疗药物、靶向药物及抗血管生成药物总结晚期肺鳞癌的治疗及获益。

## 1 细胞毒性药物（cytotoxic chemotherapy）

美国国立综合癌症网络指南（National Comprehensive Cancer Network, NCCN）推荐美国东部肿瘤协作组（Eastern Cooperative Oncology Group, ECOG）体能状态评分（performance status, PS）为0分-1分的晚期肺鳞癌患者应给予含铂两药化疗方案作为一线治疗。Azzoli和Reck的研究证实，标准含铂两药化疗方案总缓解率为25%-35%，中位无进展生存时间（progression-free survival, PFS）约为4个月-6个月，中位总生存期（overall survival, OS）约为8个月-10个月^[6, 7]^。有效延长了晚期肺鳞癌患者的生存时间。

在一项大型随机Ⅲ期研究^[8]^中，1, 725例Ⅲb期-Ⅳ期NSCLC患者被随机分到培美曲塞+顺铂（*n*=862）及吉西他滨+顺铂组（*n*=863），最终两者OS无明显差异，但是在亚组分析中，对于肺腺癌患者，培美曲塞+顺铂组明显优于吉西他滨+顺铂组，中位OS分别为11.8个月和10.4个月（HR=0.81, 95%CI: 0.70-0.94）；而在鳞癌患者中正好相反，培美曲塞+顺铂组总生存劣于吉西他滨+顺铂组（HR=1.23, 95%CI: 1.00-1.51）。Liao等的研究^[9]^指出：包括紫杉醇、多西他赛、吉西他滨或长春瑞滨在内的含铂两药标准一线化疗方案在肺鳞癌患者中总生存时间相同。最新的NCCN指南也延续了以往的观点，将紫杉醇、多西他赛、吉西他滨或长春瑞滨联合铂类双药作为肺鳞癌的标准一线治疗。此外，一项Ⅲ期临床对照研究显示，在肺鳞癌患者中，白蛋白紫杉醇+卡铂对比传统紫杉醇+卡铂，具有更高的总缓解率（41% *vs* 24%, *P*＜0.001），且毒副作用发生率低^[10]^。另外一项随机Ⅱ期临床对照研究也证实了上述观点，该研究纳入了120例既往未接受过治疗的晚期肺鳞癌患者，按照1:1的比例分别接受白蛋白紫杉醇+卡铂和吉西他滨+卡铂治疗，结果显示，虽然总生存相同，但前者的客观缓解率高（40% *vs* 19%）^[11]^。

替吉奥（S-1）是一种由替加氟、吉莫斯特和氧嗪酸钾3种成分按照摩尔比1:0.4:1组成的口服的氟尿嘧啶衍生物^[12]^。一项随机Ⅲ期临床研究显示，S-1作为晚期肺鳞癌一线治疗方案效果显著。该研究纳入了564例晚期肺鳞癌患者，S-1+卡铂组对比安慰剂组的中位OS分别为14.0个月和10.6个月（HR=0.713, 95%CI: 0.476-1.068）^[13]^。

尽管接受了含铂两药的一线化疗后，肿瘤不可避免的都会出现进展。对于一线治疗失败后，NCCN指南推荐多西他赛、培美曲塞和厄洛替尼为NSCLC的标准二线治疗方案。而对于肺鳞癌患者，多西他赛优于培美曲塞和厄洛替尼^[14]^。但是由于多西他赛显著的毒性反应，临床应用大多受到限制^[15]^。近期一项关于晚期肺鳞癌二线治疗的研究表明，针对既往化疗失败的老年肺鳞癌患者，白蛋白紫杉醇安全有效（中位PFS 6.6个月，中位OS 10.9个月）^[16]^。

## 2 靶向治疗

对于晚期或者转移的NSCLC患者，FDA批准EGFR抑制剂为*EGFR*突变型的一线化疗药物。包括第一代EGFR抑制剂：吉非替尼和厄洛替尼，第二代EGFR抑制剂：阿法替尼。但是不同于在肺腺癌中*EGFR*的高突变率，肺鳞癌患者的*EGFR*突变率往往小于5%^[17]^。来自Shukuya等^[18]^的研究指出，*EGFR*突变的NSCLC患者接受吉非替尼治疗，根据病理类型不同，鳞癌和腺癌的客观缓解率分别为36%和69%，PFS分别为3.1个月和9.4个月。但值得肯定的是，肺鳞癌*EGFR*突变型的总反应率明显高于野生型，分别为25%和9%^[19]^。在肺鳞癌中*ALK*突变率虽然较*EGFR*突变率更低，仍旧有一些个案报道^[20]^。因此，国际肺癌研究协会推荐对于年轻、不吸烟的鳞癌患者应该接受*EGFR*突变和ALK扩增检测^[21]^。来自Shepherd等^[22]^的一项随机Ⅲ期临床研究表明，尽管在NSCLC中厄洛替尼组对比安慰剂组，可延长PFS（2.2个月*vs* 1.8个月，*P*＜0.001）及OS（6.7个月*vs* 4.7个月，*P*＜0.001），但在鳞癌患者中，两者的OS没有明显统计学差异（*P*＞0.05）。来自Kim等^[23]^的临床研究也证实了这个观点，在二线肺鳞癌患者中吉非替尼对比多西他赛，差异没有统计学意义。但是，值得关注的是，同样在肺鳞癌的二线治疗中，阿法替尼与厄洛替尼的中位PFS分别为2.4个月和1.9个月（*P*＜0.05），中位OS分别为7.9个月和6.8个月（*P*＜0.05），有明显的统计学差异^[24]^。

AZD9291是第三代EGFR抑制剂，它的出现给既往EGFR-TKI耐药的患者带来了新的希望。该药是一种不可逆的EGFR抑制剂，选择性抑制*EGFR*敏感突变和T790M耐药突变。多项临床研究表明，AZD9291对于EGFR抑制剂耐药者，无论是T790M阳性组还是阴性组，都能取得很好的疗效，其中阳性组尤其明显^[25, 26]^。

单克隆表皮生长因子受体抑制剂如西妥昔单抗和Necitumumab在NSCLC治疗中也取得了不错的进展。一项Ⅲ期随机临床研究表明：西妥昔单抗对比长春瑞滨联合顺铂方案治疗肺鳞癌患者具有细微的生存获益（*P*=0.044）^[27]^。另外，美国礼来公司对Necitumumab进行了一项开放、随机化、多中心Ⅲ期试验，结果显示Necitumumab联合吉西他滨和顺铂治疗组与单纯吉西他滨和顺铂治疗组相比可以明显延长晚期肺鳞癌患者的总生存，中位OS分别为11.5个月和9.9个月（*P*=0.012）^[28]^。

## 3 抗血管生成药物

血管内皮生长因子（vascular endothelial growth factor, VEGF）在肿瘤血管生成过程中起着关键作用，已成为抗肿瘤治疗的重要靶点。贝伐珠单抗（Bevacizumab）是一种VEGF-A抑制剂。在一项随机的Ⅱ期临床试验中，99例晚期NSCLC患者随机接受贝伐珠单抗+卡铂+紫杉醇和卡铂+紫杉醇治疗，二者中位PFS分别为7.4个月和4.2个月，中位OS分别为17.7个月和14.9个月。但是该药有严重的毒性反应，如肺出血、肺栓塞，在肺鳞癌中尤其突出^[29]^。雷莫芦单抗（Ramucirumab），是一种VEGFR-2抑制剂。一项随机、双盲的多中心Ⅲ期临床研究^[30]^纳入了1, 253例既往含铂类双药化疗失败后的晚期NSCLC患者，按照1:1比例，分别接受雷莫芦单抗+多西他赛方案及安慰剂+多西他赛方案用治疗，二者中位PFS分别为4.5个月和3.0个月（HR=0.762, *P*＜0.000, 1），中位OS分别为10.5个月和9.1个月（HR=0.857, *P*=0.023）。但是在肺鳞癌患者中，两者OS分别为9.5个月和2个月，无明显统计学差异（HR=0.88, 95%CI: 0.69-1.13），甚至较安慰剂组在不良事件流血/出血（28.9% *vs* 15.2%）上发生率更高。此外，2项来自中国肺癌杂志的研究表明：对于初治后进展或不能耐受毒性反应的NSCLC（包括晚期肺鳞癌）患者，VEGF抑制剂重组人血管内皮抑素（Endostar）能在不增加毒副作用的情况下延长多西他赛化疗获益患者的疾病进展时间^[31, 32]^。

## 4 总结

肺癌仍旧是目前世界上发病率和死亡率最高的恶性肿瘤之一。2012年《世界癌症报告》指出，2012年因肺癌去世人数约为159万。肺鳞癌作为其中最常见的肺癌类型之一，经手术、放化疗等综合治疗后，其5年生存率仍低于15%^[33]^。治疗上，含铂双药仍为晚期肺鳞癌患者一线标准化疗方案，但是除紫杉醇、多西他赛、吉西他滨或长春瑞滨以外，白蛋白紫杉醇、S-1联合铂类也取得了显著的疗效。此外，推荐对于年轻、不吸烟的患者可以进行*EGFR*及*ALK*基因检测，如果突变阳性，推荐使用相应的分子靶向药物。一线治疗失败后，除多西他赛外，白蛋白紫杉醇、阿法替尼等多种药物在肺鳞癌方面的治疗优势也被渐渐显露出来。值得高兴的是，如纤维母细胞生长因子受体1（fibroblast growth factor receptor 1, FGFR1）、盘状结构域受体2（discoidin domain receptor 2, DDR2）、胰岛素样生长因子1受体（insulin-like growth factor 1 receptor, IGF-1R）和磷脂酰肌醇-3-激酶催化亚单位α（phosphoin-3-kinase catalytic alpha polypeptide, PIK3CA）等^[34]^关于肺鳞癌的潜在靶点药物正在发展。来自细胞程序性死亡蛋白1（programmed cell death protein 1, PD-1）/程序性死亡因子配体1（programmed cell death protein ligand 1, PD-L1）抑制剂的一些研究结果已经证实，与多西他赛相比，PD1抑制剂Nivolumab在晚期肺鳞癌患者中客观缓解率更高、毒副反应更低，且能延长PFS及OS^[35, 36]^。FDA批准了Nivolumab用于治疗在以铂类基础化疗期间或化疗后发生疾病进展的转移性肺鳞癌。综上所述，化疗药物的发展以及基于肺鳞癌靶基因的靶向治疗药物及免疫治疗的出现，希望能给晚期肺鳞癌患者带来更多获益。
